# Supplementary material for: Cerebral mGluR5 availability contributes to elevated sleep need and behavioral adjustment after sleep deprivation
Source: eLife. 2017 Oct 5;6:e28751. doi: 10.7554/eLife.28751 (PMC5644949; doi:10.7554/eLife.28751)
Supplement: Transparent reporting form [file elife-28751-transrepform.docx]

***eLife’s* transparent reporting form**

We encourage authors to provide detailed information *within their submission* to facilitate the interpretation and replication of experiments. If you have any questions, please contact us: [editorial@elifesciences.org](mailto:editorial@elifesciences.org).

**Sample-size estimation**

- You should state whether an appropriate sample size was computed when the study was being designed
- You should state the statistical method of sample size computation and any required assumptions
- If no explicit power analysis was used, you should describe how you decided what sample (replicate) size (number) to use

Please outline where this information can be found within the submission (e.g., page numbers or figure legends), or explain why this information doesn’t apply to your submission:

**Human studies:**

No formal statistical method was used to predetermine the sample size of the neuroimaging studies in humans. With 26 participants in the positron emission tomography [PET] study (23 could be included in data analyses) and 17 participants undergoing MR spectroscopy, the sample size was kept higher than in comparable studies in which the effects of sleep deprivation were assessed with positron emission tomography (e.g., Elmenhorst et al., *J Neurosci*, 2007 [n = 10-12] ; Volkow et al., *J Neurosci*, 2008 [n = 15]; Elmenhorst et al., *Proc Natl Acad Sci USA*, 2017 [n = 15]).

The sample sizes are described in detail on p. 5, line 119; p. 7, line 151; p. 19, line 441; p. 20, line 460; p. 21, line 482; p. 22, 523-525.

**Mouse studies:**

Standard procedures and similar sample sizes as in previous reports were used (this information is not specifically indicated in manuscript):

**- vigilance states and EEG:** n=8; sample size similar or higher than reported in other studies (e.g., Franken et al., *Sleep*, 1999, Mikhail et al., *Sci Signal*, 2017)

**- Y-maze:** n= 6-10; similar to sample sizes reported in other studies (e.g., Ramanathan et al., *Behav Brain Res*, 2010; Hagewoud et al., *Sleep*, 2010)

**- qPCR and *Grm5* mRNA expression:** n= 4 biological replicates and n=3 technical replicates, standard method as described before (e.g., Mikhail et al., *Sci Signal*, 2017)

**Replicates**

- You should report how often each experiment was performed
- You should include a definition of biological versus technical replication
- The data obtained should be provided and sufficient information should be provided to indicate the number of independent biological and/or technical replicates
- If you encountered any outliers, you should describe how these were handled
- Criteria for exclusion/inclusion of data should be clearly stated
- High-throughput sequence data should be uploaded before submission, with a private link for reviewers provided (these are available from both GEO and ArrayExpress)

Please outline where this information can be found within the submission (e.g., page numbers or figure legends), or explain why this information doesn’t apply to your submission:

**Human studies:**

All human data were collected in a highly controlled, supervised, randomized, cross-over, sleep deprivation protocol. All subjects served as their own controls (within-subjects design, biological replicates). Data was collected only once.

Information on data collection and outliers are described in detail in the Results and Methods sections: p. 7, lines 153-156; p. 19, lines 441-446; p. 20, lines 457-461; p. 22, lines 519-526.

**Mouse studies:**

***Grm5* mRNA expression:**

- Biological replicates: n=4 mice per group for both qPCR experiments, i.e. mRNA extraction from whole brain and dissected brain areas. Some RNA extracts of dissected brain areas did not fulfill the stringent quantity and quality requirements. A total of n =3 samples per condition and brain area was analyzed. Results main text (p. 8, line 178), Legend to Fig. 5 (p. 32) and Methods section (p. 27, lines 642-647)

- Technical replicates: n=3 per sample. Methods section (p. 27, line 654).

- Inclusion/exclusion criteria: RNA quantity (> 1 µg) and quality (260/280 ~2.0) control by NanoDrop. No template control: PCR negative control. No enzyme control: DNA contamination control. Outlined in Methods section (p. 27).

**qPCR:**

- Biological replicates: n=4 mice per genotype: Results main text (p. 8, line 181) and Methods section (pp. 27/28).

- Technical replicates including definition: n= 3: Methods section (p. 27, lines 650-653).

**Vigilance states and EEG:**

- Biological replicates: n=8 mice per group: Legend to Fig. 6 (p. 33) for amount of vigilance states (sleep rebound) and Methods section (p. 24-25, lines 570-571 & 594).

- Biological replicates: n=7-8 mice per group: Legend to Fig. 7 (p. 33) for EEG delta power time course and Methods section; EEG delta power above 2 standard deviations of the mean in one mouse during recovery → excluded from analysis (p. 27, lines 635-639)

- Inclusion/exclusion criteria: values < / > 2 standard deviations from the mean were considered as outliers and excluded from analyses.

**Y-Maze:**

- Biological replicates: n=6-10 mice per group: Legend to Fig. 8 (p. 34) and detailed number of mice in each group explained in Methods section (p. 28, lines 684-685).

- Description of the Y-Maze protocol: Results (pp. 11-12) and Methods sections (pp. 28/29).

- Inclusion/exclusion criteria: spontaneous exploration in the maze.

**Statistical reporting**

- Statistical analysis methods should be described and justified
- Raw data should be presented in figures whenever informative to do so (typically when N per group is less than 10)
- For each experiment, you should identify the statistical tests used, exact values of N, definitions of center, methods of multiple test correction, and dispersion and precision measures (e.g., mean, median, SD, SEM, confidence intervals; and, for the major substantive results, a measure of effect size (e.g., Pearson's r, Cohen's d)
- Report exact p-values wherever possible alongside the summary statistics and 95% confidence intervals. These should be reported for all key questions and not only when the p-value is less than 0.05.

**Human studies:**

Statistical analyses methods, number of subjects, description, and correction for multiple comparisons are described in the Methods section and in the legends to the figures.

**PET and EEG data:** p. 29, lines 700-704; legends to Figs. 2 and 3 (pp. 31/32). The correlation analyses in Figs. 2 and 3 illustrate individual data, i.e., each point in the figures represents a single subject.

For the regional correlations with < 1 Hz EEG activity, all correlations are reported in supplementary Table S1. Significant effects surviving Bonferroni correction for multiple comparisons are indicated with a star.

**MRS and PET data:** p. 22, lines 522-526; p. 10, legend to Fig. 4 (p. 32). The correlation analysis in Fig. 4 illustrates individual data, i.e., each point in the figure corresponds to a single subject.

**Mouse studies:**

Because the Figs. 6-8 are already complex, means and standard deviations are depicted rather than raw values to ensure readability and comprehensibility.

**qPCR:**

- Statistical analysis method and justification, n value: Results (p. 8) and Methods sections (p. 27/28)

- Definition of center and dispersion measure, exact p-values: Results main text (p. 8)

***Grm5* mRNA expression:**

- Statistical analysis method description and justification, n values, method of multiple test correction: see Methods section (p. 27)

- Definition of center and dispersion, exact p-values: Results (p. 8) and legend to Fig. 5 (p. 32)

**Vigilance states and EEG:**

- Statistical analysis method description and justification, n values, method of multiple test correction, definition of center and dispersion, exact p-values: Results (pp. 8-10); Legends to Fig. 6 (p. 33) and figure supplements 1 and 2; Legend to Fig. 7 (p. 33); Methods section (pp. 25-27).

Please outline where this information can be found within the submission (e.g., page numbers or figure legends), or explain why this information doesn’t apply to your submission:

**Ymaze:**

- Statistical analysis method description, exact n values, method of multiple test correction, definition of center and dispersion exact p-values: Results (pp. 11-12); Legend to Fig. 8 (pp. 34) and figure supplement 1; Methods section (pp. 28/29).

(For large datasets, or papers with a very large number of statistical tests, you may upload a single table file with tests, Ns, etc., with reference to page numbers in the manuscript.)

**Additional data files (“source data”)**

- We encourage you to upload relevant additional data files, such as numerical data that are represented as a graph in a figure, or as a summary table
- Where provided, these should be in the most useful format, and they can be uploaded as “Source data” files linked to a main figure or table
- Include model definition files including the full list of parameters used
- Include code used for data analysis (e.g., R, MatLab)
- Avoid stating that data files are “available upon request”

Please indicate the figures or tables for which source data files have been provided:

**Human studies:**

The ethical approval granted to the authors by the IRB/ethics committee does not allow the publication of the raw data online. If readers would like to re-analyze the data set (for different purposes), additional ethical approval (on an individual user and purpose basis) will be required. The authors would be happy to support additional ethical approval applications from researchers for access to the data set.

**Mouse studies:**

Tables with the source data of Figs. 5-8 will be provided:

- *Grm5* mRNA expression

- accumulated differences in vigilance states per hour and mouse

- time course of vigilance states

- time course of EEG delta power

- spontaneous alternation behavior

- exploratory activity
